# Interaction with a Hand Rehabilitation Exoskeleton in EMG-Driven Bilateral Therapy: Influence of Visual Biofeedback on the Users’ Performance

**DOI:** 10.3390/s23042048

**Published:** 2023-02-11

**Authors:** Ana Cisnal, Paula Gordaliza, Javier Pérez Turiel, Juan Carlos Fraile

**Affiliations:** 1 Instituto de las Tecnologías Avanzadas de la Producción (ITAP), School of Industrial Engineering, University of Valladolid, 47011 Valladolid, Spain; 2 Basque Center for Applied Mathematics (BCAM), 48009 Bilbo, Spain

**Keywords:** biofeedback, electromyography, human–robot interaction, neuromotor rehabilitation, robotics

## Abstract

The effectiveness of EMG biofeedback with neurorehabilitation robotic platforms has not been previously addressed. The present work evaluates the influence of an EMG-based visual biofeedback on the user performance when performing EMG-driven bilateral exercises with a robotic hand exoskeleton. Eighteen healthy subjects were asked to perform 1-min randomly generated sequences of hand gestures (rest, open and close) in four different conditions resulting from the combination of using or not (1) EMG-based visual biofeedback and (2) kinesthetic feedback from the exoskeleton movement. The user performance in each test was measured by computing similarity between the target gestures and the recognized user gestures using the L2 distance. Statistically significant differences in the subject performance were found in the type of provided feedback (*p*-value 0.0124). Pairwise comparisons showed that the L2 distance was statistically significantly lower when only EMG-based visual feedback was present (2.89 ± 0.71) than with the presence of the kinesthetic feedback alone (3.43 ± 0.75, *p*-value = 0.0412) or the combination of both (3.39 ± 0.70, *p*-value = 0.0497). Hence, EMG-based visual feedback enables subjects to increase their control over the movement of the robotic platform by assessing their muscle activation in real time. This type of feedback could benefit patients in learning more quickly how to activate robot functions, increasing their motivation towards rehabilitation.

## 1. Introduction

The incidence of stroke is growing because of the ageing population. Although the stroke mortality has been reduced, the increase of stroke survivals has resulted in an increasing number of adults with disabilities, and therefore the demand for stroke rehabilitation services is also growing [1]. This aspect has elicited considerable scientific interest in motor recovery using robotic rehabilitation systems.

Robotic assistance devices are deployed in clinical settings as a rehabilitation tool with a special focus on arm function and gait [2]. There are two main categories based on their design [3]: end-effectors [4,5] and exoskeletons [6,7,8]. Additionally, they can also be classified according to training modality or assistance type: passive, assistive, active or resistive [9].

As opposed to passive robots that always guide the movement of the paretic limb, assistive robots only exert assisting forces if the patient intends to do the movement. Both passive and assistive modes are aimed to be used in the first stages of stroke rehabilitation, when the patient has not enough strength to move the paretic limb, while active and resistive modes are used in later phases because they require patient movement.

The passive training modality has been found to be temporarily effective for reducing hypertonia [10] and for maintaining the range of motion in the early stage of treatment but it does not significantly improve motor functionality [11]. Assistive rehabilitation robots have been found to be more effective for motor skills improvement than passive robots [11,12]. However, the design complexity of assistance robots increases when it is necessary to detect the movement intention of the patient.

Hence, enabling a natural human–robot interaction (HRI) is a major challenge in developing robotic rehabilitation systems. The selected strategy for intention detection is crucial for a transparent and friendly HRI. Some robotic rehabilitation platforms use bio-signals as an intention recognition source. Electromyographic (EMG) signals are most commonly used since they are closely related to the human motion [13,14,15]: they represent the electrical activity produced by the skeletal muscles responsible for performing the intended gestures and actions [16,17,18]. Although the accuracy of the intention detection strategy is mostly related to the sensors and algorithms [19], several studies have reported the influence of the learning effect on the user’s performance after repeated used of the robotic platform [20]. Hence, providing some feedback about EMG activity may help the user to learn more quickly how to control the robot because it may improve the user’s motor control. This way, the user’s learning time may be shorter and, consequently, their motivation may be enhanced.

The biofeedback approach was introduced more than forty years ago in rehabilitation settings [21]. It consists of providing the user with information about their physiological activity in real time that would otherwise be unknown. EMG biofeedback is the most widely used method of biofeedback and it is usually provided to the user by visual or auditory signals [22,23]. These are known as EMG-based visual feedback and EMG-based audio feedback, since the input source is based on EMG information and is fed back to the user visually and auditorily, respectively. Although EMG biofeedback techniques appear promising, there is limited and contradictory evidence about their effectiveness in musculoskeletal and neurological rehabilitation [24,25,26]. However, studies that tried to assess the effectiveness of EMG biofeedback did not use this technique in combination with robotic rehabilitation platforms [27,28,29,30,31,32,33,34,35,36,37,38,39,40,41,42,43,44,45,46,47,48,49,50,51,52].

For this reason, we aim to evaluate whether the inclusion of EMG biofeedback enables users to gain control over the movement of EMG-driven robotic rehabilitation platforms by better assessing their EMG responses and, therefore, to learn self-control of these responses. For this assessment, the RobHand platform, an EMG-driven robotic hand exoskeleton for performing bilateral neuromotor rehabilitation therapies, is used. 

EMG-driven bilateral therapies consist of recording the muscle activity of the healthy limb to determine the motion and to reproduce it in the paretic limb with the assistance of the robotic device. Bilateral robotic therapies follow the same principles of the traditional mirror therapy with the only difference that the motion illusion created by the mirror is replaced by the real motion provided by the exoskeleton. EMG-driven bilateral therapies are included in the assistive training modality. Hence, they have great potential during the first weeks of rehabilitation when the motor capabilities are limited, but also because the muscle activity of the impaired limb may be unreliable for unilateral control.

During the experiments, EMG information was visually fed back to the user while undergoing EMG-driven bilateral exercises. Furthermore, the EMG-based visual biofeedback was designed specifically for this study with the aim of being as simple as possible using a visualization based on two variable length bars and two colors. Its effectiveness was investigated by comparing the user performance with and without the presence of this feedback. Furthermore, we investigated whether the kinesthetic feedback from the exoskeleton movement also induced an enhancement over the robot control.

## 2. Materials and Methods

### 2.1. Participants

The study was conducted with 18 subjects, all legal age (mean age was 23 ± 3.4) and students from the University of Valladolid. All subjects were healthy, with no neurological or orthopedic impairment, and volunteered to participate in the study by providing written informed consent. None of the subjects had previously used EMG-driven robotic devices. The study was undertaken during the month of January 2022, using the RobHand rehabilitation platform.

### 2.2. Robot Rehabiliation Platform

**Figure 1:**
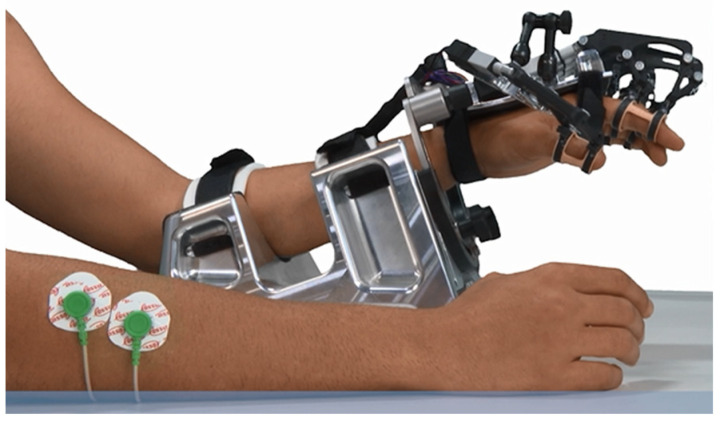
RobHand exoskeleton.

The RobHand (Robot for Hand rehabilitation) robotic neurorehabilitation platform is based on a hand exoskeleton that allows performing of EMG-driven bilateral therapies (Figure 1). The exoskeleton assists the hand fingers in the flexion and extension movements, and it is based on a direct-driven under-actuated serial four-bar linkage mechanism. Specifically, the hand exoskeleton has a range of motion of the metacarpophalangeal (MCP) joint of 2~−78°, which indicates that flexion of the finger reaches −72°, and the extension reaches 2° [53].

The EMG-driven bilateral therapies are carried out by recognizing the gesture of the healthy hand (open, rest or close) by analyzing the EMG signals and replicating that gesture on the hand exoskeleton, which is placed on the paretic hand. For this purpose, the electromyographical signals of the extensor digitorum (ED) and flexor digitorum (FDS) muscles of the healthy hand are recorded by a custom-made 2-channel low-cost EMG acquisition system. Each channel consists of an instrumentation amplifier and a RC low-pass filtered. ED and FDS are two of the muscles responsible for the hand opening and closing movements, respectively. The EMG signals are recorded at a sampling rate of 200 Hz and are processed in the real-time TMS320F28069M microcontroller (Texas Instruments, Dallas, TX, USA) to detect the hand gestures. 

The hand gesture recognition is performed by an EMG-based threshold algorithm. Hence, it is performed by muscle activity comparison, which requires a previous normalization of the EMG signals; the raw signals must be mapped into a range of 0–1, implying a previous rectification of the signal. The 0 value stands for no muscle activity whereas the value 1 stand for the maximal voluntary contraction (MVC) of the muscle.

Therefore, the recorded EMG signals are notch-filtered at 50 Hz and high-pass filtered at 10 Hz to isolate the spectral content of interest. The filtered signals are rectified by calculating the root mean square (RMS) with a moving average window of 50 ms (10-point window) and then low-pass filtered at 2 Hz. The rectified signals (rEMG), with a resulting frequency of 20 Hz, are normalized with respect to the MVC values, which have been determined in a previous calibration. The gesture recognition depends on the normalized signals (nEMG) of the ED and FDS muscles and the two EMG thresholds, which have also been calculated in previous calibrations [54,55]. 

**Figure 2:**
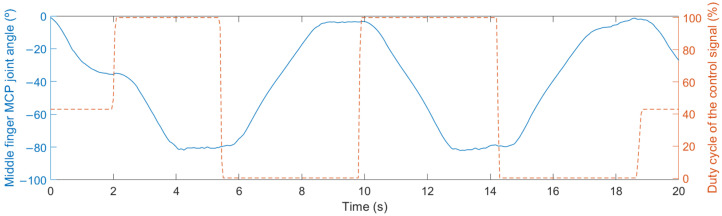
Recorded data of the MCP joint angle of the middle finger and duty cycle of the PWM control signal applied to the actuator.

Lastly, the microcontroller generates the proper control signals to move the L12-30-100-6-I actuators (Actuonix Motion Devices Inc., Saanichton, BC, Canada) of the hand exoskeleton to the target position. The control signal is a PWM signal, whose duty cycle is proportional to the stroke extension percentage of the actuator. For instance, Figure 2 shows that when the duty cycle changes to 0% the actuator starts moving so the exoskeleton reaches the closed gesture (MCP joint angle of −78°). Similarly, when the duty cycle changes to 100%, the actuator starts moving in the way of opening the hand exoskeleton (MCP joint angle of 2°) [56]. Further details regarding the electronic design and operation of the real-time system, including the custom-made EMG acquisition system, microcontroller and actuators can be found in Cisnal et al. [54].

#### 2.2.1. Calibration Process

**Figure 3:**
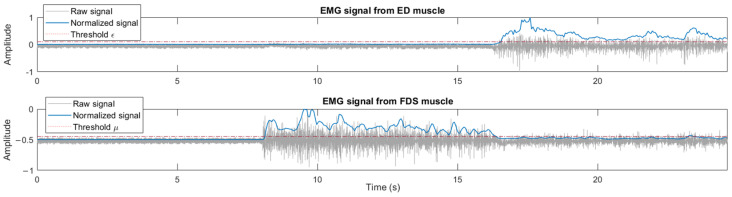
EMG signals of ED and FDS muscles during a calibration process: raw EMG signals, normalized EMG signals and deactivation thresholds.

The calibration process is required to determine both the MVC values for normalizing the signals and the muscular deactivation thresholds used in the EMG-driven control. The duration of the calibration is 24 s: subjects are asked to rest their hand and to perform a maximum hand finger flexion and extension for 8 s for each action (Figure 3).

The MVC of the ED and FDS muscles (MVC_ED_ and MVC_FDS_) are determined as the maximum value of their corresponding rectified EMG signal (rEMG) during the calibration procedure. The two thresholds, ε and µ, correspond to the muscular deactivation of the ED and FDS muscles. Therefore, the thresholds are determined as the minimum value of their corresponding normalized EMG signals (nEMG) plus a constant of 0.1 during the relaxation phase [54].

#### 2.2.2. EMG-Driven Control

**Figure 4:**
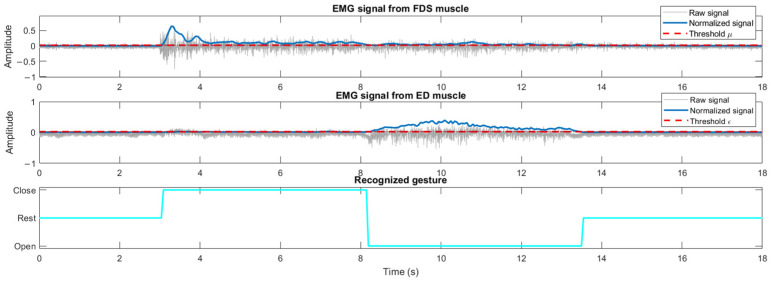
Gesture recognition using the threshold EMG-driven control: raw EMG signals of ED and FDS muscles, normalized EMG signals, thresholds and determined gestures.

The EMG-driven control recognizes the actual gesture of the healthy hand and replicates it on the hand exoskeleton. Three gestures can be recognized: rest, open and close. The recorded EMG signals are transmitted to the real-time microcontroller to filter, rectify and normalize the signals. Then, the gesture is recognized according to the normalized EMG signals (nEMG) and the two EMG thresholds (ε and µ) set in the initial calibration (Figure 4). The rest gesture is determined when both nEMG signals are lower than their respective muscular deactivation thresholds. The open-hand gesture is recognized when the nEMG_ED_ signals exceed the extensor threshold (ε) and the nEMG_ED_ is larger than nEMG_FDS_ if nEMG_FDS_ exceeds the flexor threshold (µ). Analogously, the close gesture is recognized when the nEMG_FDS_ signal exceeds the flexor threshold (µ) and the nEMG_FDS_ is larger than nEMG_ED_ if it exceeds the extensor threshold (ε). 

Formally, if A is defined to be true when nEMG_ED_ is higher than ε, B is true when nEMG_FDS_ is higher than µ and C is true when nEMG_ED_ is higher than nEMG_FDS_, the gesture recognition algorithm can be defined by the following Equations (1)–(3).

(1) $$\mathrm{REST}=\bar{A}\cdot \bar{B}$$

(2) $$\mathrm{OPEN}=A\cdot \left(\bar{B}+C\right)$$

(3) $$\mathrm{CLOSE}=B\cdot \left(\bar{A}+\bar{C}\right)$$

Note that for any combination of inputs (A, B and C), only one of the outputs (REST, OPEN or CLOSE) is true [54].

### 2.3. Experimental Protocol

**Figure 5:**
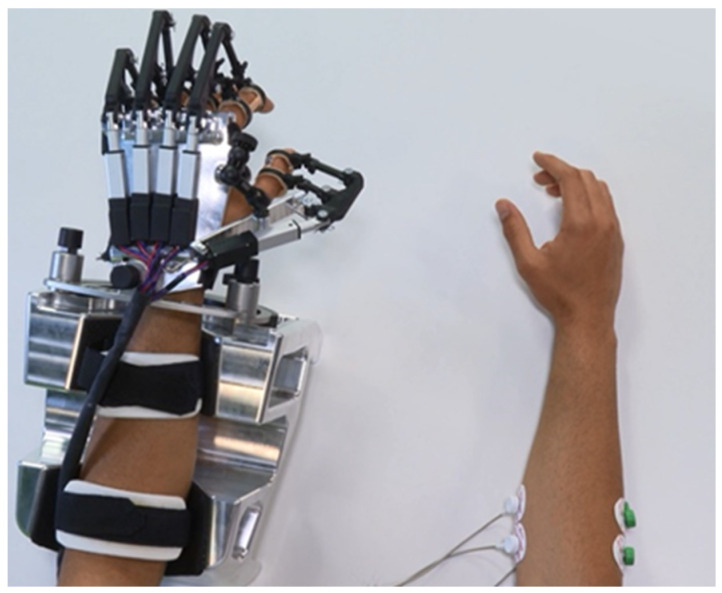
Experimental setup. Surface electrodes are connected to the dominant ED and FDS muscles, while the exoskeleton is placed in the non-dominant hand of the subject.

The subjects are comfortably seated in a chair in front of a table, looking into a computer screen positioned approximately 50 cm away. Since the experiments are carried out with healthy subjects, their dominant hand corresponds to the non-paretic hand of the patients and their non-dominant hand corresponds to the impaired hand of the patients. The positions for the placement of the EMG electrodes are determined by palpating and visually observing muscle contractions in the dominant forearm. The hand exoskeleton is placed on the non-dominant hand (Figure 5). The dominant arm is held in a self-selected comfortable position (e.g., on their leg or on the table). The principle of EMG-driven hand exoskeleton operation is explained, and the EMG calibration procedure is performed before starting the experimental trial.

All subjects perform four different experimental tests: A, B, C and D. Each test has a duration of 1 min and there is a 3-min break between tests to eliminate the effects of possible muscular fatigue. Furthermore, the four tests are randomly performed to eliminate the possible learning order effect. A test consists of performing and maintaining a hand gesture (rest, open or close) with the dominant hand following the sequence of gestures indicated by visual and sound information from a computer program. The indicated gestures are random generated, each lasting three seconds. Hence, a randomly sequence of 20 gestures are performed during each one-minute test. 

The main difference between the four tests is the presence of different sources of feedback: kinesthetic and EMG-based visual feedback. The kinesthetic feedback is provided by the movement of the hand exoskeleton, which replicates the detected hand gesture by analyzing the EMG (Figure 2). The EMG-based visual feedback is provided by the computer screen.

Table 1 shows the feedback configuration for each test. In tests A and B, the hand exoskeleton is operative and moves the non-dominant hand of the subject according to the analysis of the EMG signals collected (EMG-driven control). Hence, there is kinesthetic feedback due to the exoskeleton’s own motion in the tests A and B. On the contrary, in tests C and D, the hand exoskeleton is not operative and, therefore, it does not make any movement, remaining continuously in the resting position. In addition, in tests A and C, the EMG-based visual feedback is visible on the computer screen, while this is hidden in tests B and D. 

**Figure 6:**
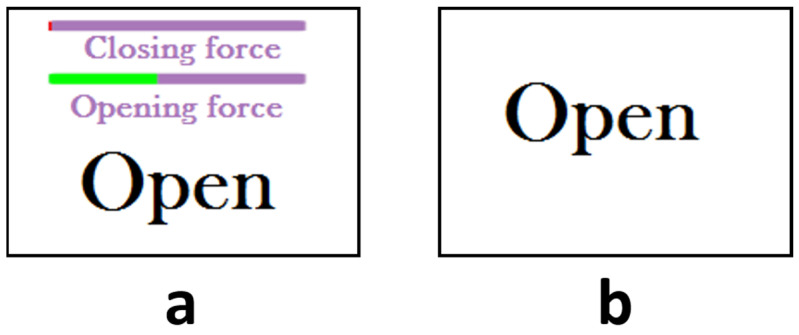
Computer screen indicating the gesture to be performed and (**a**) with or (**b**) without EMG-based visual feedback.

The EMG-based visual feedback comprises two variable length bars, whose lengths represent the instantaneous value of the normalized signals from the ED and FDS muscles (nEMG_ED_ and nEMG_FDS_). The bars are labeled as ‘Opening force’ and ‘Closing force’ so that the user can easily understand what they mean. The bars change color to indicate the recognized gesture according to Equations (1)–(3). The bars turn red or green to indicate whether the gesture recognition module has detected that the hand is at rest (both bars are red), is opened (opening force bar is green while closing force bar is red) or is closed (opening force bar is red while closing force bar is green). As previously said, the computer screen incorporates the gesture to perform by the user. Figure 6 shows the configuration of the computer screen with and without EMG-based visual feedback.

**Figure 7:**
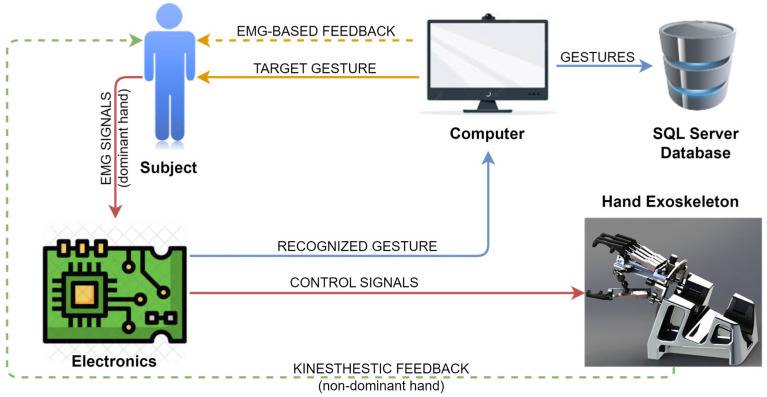
Experimental protocol setup. Visual information, signals, data transmission and movement are indicated by yellow, red, blue, and green lines, respectively. Dotted lines indicate the source of feedback, which may or may not be provided to the subject.

The overall system setup for the experimental protocol is shown in Figure 7. The subject is asked to perform a gesture (open, close or rest hand) with their dominant hand by visual and audio indicators provided by the computer. The target gesture is randomly generated every three seconds. Meanwhile, EMG signals from the ED and FDS muscles of the subject are recorded by a custom-made EMG acquisition system at 200 Hz. Then, the EMG signals are transmitted to the microcontroller to recognize the performed gesture and to generate control signals to move the exoskeleton’s actuators accordingly. The normalized EMG signals and the recognized gestures are transmitted to the PC over USB at 20 Hz (T_s_ = 0.05 s), which is the resulting sampling frequency of the normalized EMG signals and, hence, the recognized gesture is updated at that frequency. The UART module of the microcontroller was set to transmit data at a rate of 115,200 bits per second (bps). Both the recognized and the target gestures are registered and stored in a SQL database as a temporal series for offline analysis at 20 Hz. The length of the bars of the EMG-based visual feedback are also updated at that same frequency. Finally, EMG-based visual feedback and kinesthetic feedback from the movement of the hand exoskeleton are provided or not depending on the type of test (test A, B, C or D).

**Figure 8:**
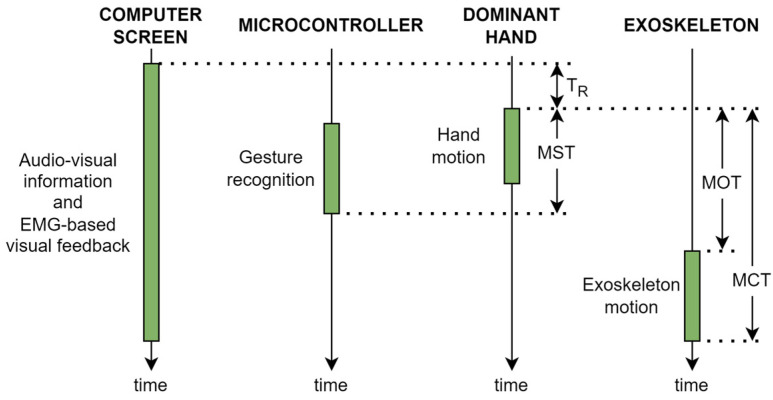
Timing diagram of the experimental procedure.

A timing diagram of the experimental procedure is shown in Figure 8. Audio and visual information is provided to indicate the user which gesture must be performed. Consequently, the user starts to move the dominant hand after a response time. Hence, the user response time (T_R_) is defined as the time from the beginning of the visual and audio information that indicates to the user which gesture to perform to the onset of the dominant hand movement. The motion-selection time (MST) is the time since the onset of the hand motion performed to reach the target gesture to the instant of time in which that gesture is recognized by the microcontroller after processing the muscle activity that generates that hand movement. Once the gesture is recognized, the microcontroller generates the proper control signals to move the exoskeleton’s actuators. However, due to the intrinsic characteristics of the actuators, the motion does not start immediately after receiving the position command and takes some time to reach the target gesture (see Figure 2). The motion-onset time (MOT) and the motion-completion time (MCT) are the elapsed time from the onset of the dominant hand movement to the onset and end of the exoskeleton motion, respectively. During the experiments, the EMG-based visual feedback is updated at a rate of 20 Hz with the results received from the microcontroller.

The EMG signal preprocessing, hand gesture recognition and control signal generation are performed in the real-time TMS320F28069M microcontroller programmed using C programming language. The computer software was developed in C# programming language using .NET framework and Microsoft Visual Studio. Furthermore, the database was designed using SQL Server Management Studio (SSMS) for saving the data for offline statistical analysis. Language Integrated Query (LINQ), which is a component of Microsoft .NET framework, was used to create query expressions to extract, process and save data from the developed relational database.

### 2.4. Data Analysis

**Figure 9:**
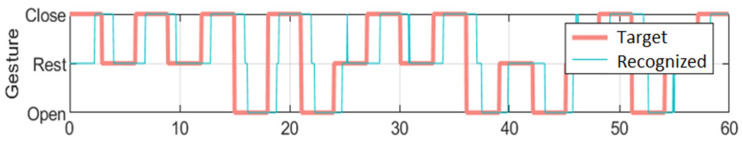
Target and recognized sequence of gestures of one test.

The target sequence of gestures performed by the user (pink line called “Target” in Figure 9) and the sequence of recognized gestures (blue line called “Recognized” in Figure 9) are saved on the system database during the experiments. Note that the target gestures are randomly updated by the computer every three seconds, while recognized gestures are identified by the threshold EMG-based algorithm (Equations (1)–(3)) running in real time at the microcontroller.

**Figure 10:**
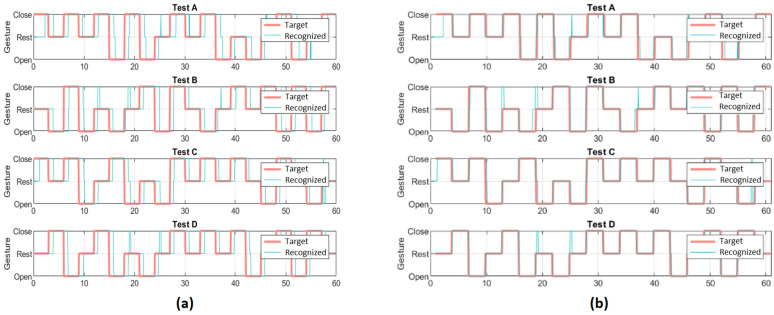
Target and recognized sequence of gestures of one individual. (**a**) Raw data; (**b**) data after synchronization.

As shown in Figure 9, the sequence of recognized gestures is delayed with respect to the sequence of target gestures. This delay is due to the user response time (T_R_) and the motion-selection time (MST). Since the performance evaluation is performed by measuring the similarity between the two discrete-event time series (Target and Recognized), for each test performed by each user, both time series are time-synchronized (Figure 10). The time synchronization is carried out using the lag at which the cross-correlation of both sequences is highest, which will be referred to hereafter as delay time (T_d_).

The cross-correlation of two time series x and y (r_xh_) is computed using Equation (4), where h is the lag and * denote the complex conjugate. Data analysis is performed using Matlab 2021a software (MathWorks) licensed to University of Valladolid.

(4) $$r_{\mathrm{xy}}\left(h\right)=\left\{\begin{array}{l} \overset{N-h-1}{\underset{n=0}{\sum }}x\left(n+h\right)\cdot y^{*}\left(n\right),\;\;0\leq h\leq N-1\\ \;\;\;\;\;\;r_{\mathrm{yx}}^{*}\left(h\right),\;-\left(N-1\right)\leq h\leq 0 \end{array}\right.$$

The performance of the subject in each test is measured by computing the similarity between the two series. Hence, the L2 distance (squared Euclidean distance) between the target gesture and the synchronized recognized gesture time series is calculated. Thus, considering the order of states “Open < Rest < Closed” (coded by “Open” = −1, “Rest” = 0 and “Close” = 1), the distance between “Open” and “Closed” is twice that of either and “Rest”. In addition, the quadratic cost penalizes the type of error more than other distances such as the L1 norm, also known as Manhattan distance, that is the sum of the absolute vector values. This is relevant because in practice a confusion between open and closed can lead to more serious consequences than either of them at rest. 

Besides, when calculating the distances between signals, it is important to consider that the synchronized signals are shorter than the reference length (one-minute test) and, moreover, that the differences between the lengths of the signals generated by each individual with each test depend on the time delay (T_d_). Therefore, the target signal is cut off at the end to make it equal in length to the synchronized signal made by the individual.

Formally, let us denote by $x={\left(x_{i}\right)}_{i=0}^{n_{\mathrm{samples}}}$ the recognized sequence for a particular individual and test, for which the time delay is T_d_ and $x_{n_{\mathrm{samples}}}=\left[60-T_{d}\right]\cdot T_{s}$, where T_s_ is the sampling period. If we denote by $x^{*}={\left(x_{i}^{*}\right)}_{i=0}^{n_{\mathrm{samples}}}$ the target signal (truncated by the end, following previous observation), then the L2 distance ($d_{L_{2}}$) between the two sequences can be computed according to Equation (5).

(5) $$d_{L_{2}}\left(x,{\;x}^{*}\right)=\sqrt{\overset{n_{\mathrm{samples}}}{\underset{i=0}{\sum }}{\left(x_{i}^{*}-x_{i}\right)}^{2}\cdot T_{s}}$$

## 3. Results

All the statistical analysis in this section is carried out using the software R (https://cran.r-project.org/, access on 5 March 2022). To detect significant effects in the performance of the subjects, a multifactorial additive ANOVA for the L2 distances of the two synchronized series is performed. The mean time delay (T_d_) for different tasks and individuals is 0.88 ± 0.14 s. The ANOVA is performed to examine the effects of the test variable, test order and individual (Table 2). Note that the consideration of individual as a factor is included in the model in order to block its effect.

**Figure 11:**
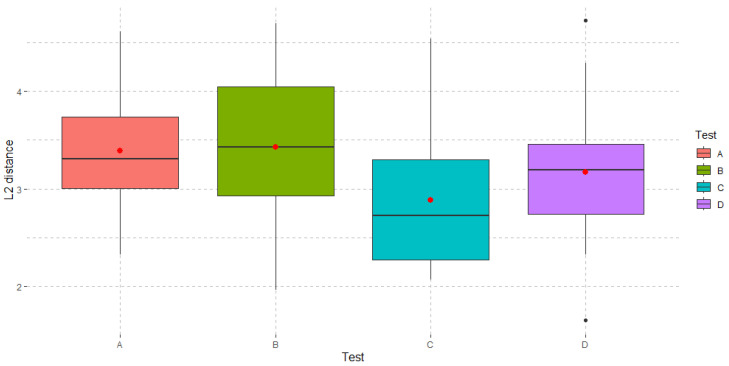
Boxplot of the L2 distances for the four performed tests.

From these results, we can conclude that there are no significant differences in the order of the tests. What is relevant is that, in terms of subject performance, significant differences are found in the type of tests taken (*p*-value 0.0124). More precisely, in Figure 11 we can see the differences in the distributions of the L2 distances conditionally given the type of test. L2 distances are 3.39 ± 0.70, 3.43 ± 0.75, 2.89 ± 0.71, 3.17 ± 0.73 for test A, B, C and D, respectively. Specifically, subjects perform better in test C than in the other tests A, B and D, the differences between the latter three not being statistically significant.

This can also be seen through the pairwise comparisons using Duncan’s multiple range test (Table 3). Importantly, the results of such a test show that the performance of the individual at test C is significantly better than at test B (*p*-value 0.038) and test A (*p*-value 0.051). Finally, all the results are supported by the fact that homoscedasticity has been checked.

## 4. Discussion

In the present study, performance of the subjects is better in test C. Thus, EMG-based visual feedback has enhanced the motor control of the user and has significantly improved accuracy during the trials. This feedback allows the subjects to monitor their EMG activation levels during the tests and compare them with the activation thresholds predefined in the previous calibration in a simple way. This enables subjects to regulate their EMG activity with respect to these threshold levels and to control the movement of the hand exoskeleton better.

On the other hand, the kinesthetic feedback does not provide significant improvement in the performance of the subjects. Neither does it improve when both feedbacks are present. This result may be related to the fact that subjects do not need to consciously pay attention to kinesthetic activity, as it is more straightforward and intuitive than the visual EMG feedback. 

**Figure 12:**
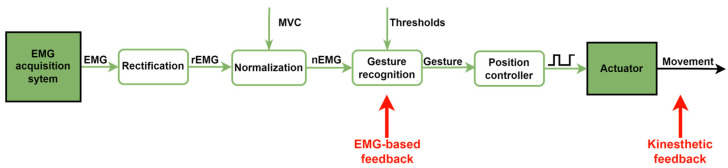
Control loop for the threshold EMG-driven control of the RobHand indicating the source of each feedback.

The main factor behind these findings is the instant of time at which each feedback modality is provided to the user. The EMG-driven control of the RobHand robotic platform works as follows (Figure 12): the recorded EMG signals are rectified (rEMG) and normalized (nEMG). The gesture recognition module determines the hand gesture based on the values of the normalized signals and the thresholds calculated in the calibration. The position controller generates the control signal related to the detected gesture so that the actuators move to reach that gesture. Hence, EMG-based visual feedback provided to the user is earlier in time than the kinesthetic feedback. Therefore, the performance of the subject is better when he/she is provided with the visual feedback as it has a longer reaction time than when the feedback is kinesthetic.

In fact, the real-time visual EMG feedback allows the user to modulate the exerted force at that very moment by directly influencing the position controller input. In contrast, with the kinesthetic feedback, the user modulates the exerted force once he/she has felt the movement performed by the actuators of the exoskeleton so that the force modulation is not immediate.

Furthermore, the hand motion generation process is not instantaneous. The motion is achieved by muscle contraction and this motor control signal is delivered from the central neural system. For any intended motor action that implies muscle contractions, it is well known that there is a time delay between the onset of the EMG signal and the onset of force production. This time delay is known as the electromechanical delay (EMD) and is about 10–300 ms [57,58]. 

In addition, the electromechanical characteristics of the actuators should be also considered: (1) the dynamic response (time interval from the instant the actuator receives a position command to the onset it starts to move) is low; and (2) the speed is not high due to the type of application (rehabilitation), with a maximum no-load speed of 12 mm/s. The time delays of the RobHand system that are considered relevant to the human–robot interaction have been determined [54]: the average of the motion-selection time (MST) and motion-onset time (MOT) are 0.48 ± 0.59 s and 0.55 ± 0.6 s, respectively. The average of the motion-completion time (MCT) is 1.90 ± 1.65 s, varying from 0.98 s (close to rest movement) to 3.42 s (open to close movement).

When the visual feedback is present, the subject modulates their force from the data of the gesture recognition module (nEMG signals and detected gesture) and anticipates the exoskeleton movement response. However, with the kinesthetic feedback the user modulates their force once the action has been performed by the exoskeleton. If the user perceives that the movement performed by the exoskeleton does not correspond to their intention, the subject can modulate their muscle activity to correct it, but it takes much longer than if he/she had corrected it based on the real-time EMG visual feedback.

Inferences in this study are based on differences in performance with and without the two proposed feedbacks. There are some limitations of the current study in the following aspects. To obtain reliable EMG recordings, standardized electrode positioning is used. However, the surface electrodes placement has a direct influence on the performance. Another limitation of the present study is the possibility of muscular fatigue during the trials, which will deteriorate the user’s performance. Three-minute breaks are included between tests to avoid this. 

It is possible, although highly unlikely due to the low number of repetitions, that a learning effect on the user performance could appear during the trials. No statistically significant difference is observed in the results as a function of the order in which the tests are performed. 

For the calculation of the L2 distance that is used to performance evaluation, it is necessary to synchronize the target and recognized time series. The time delay between these two signals has been considered constant throughout each test, although in may vary between gestures.

Experimental trials have been performed on healthy subjects, so they cannot be extrapolated to patients who have suffered damage affecting their cognitive abilities. The patient with impaired cognition and perception may become confused and distracted with the EMG-based visual biofeedback, resulting in deterioration of test performance. 

Furthermore, the EMG monitorization is carried out in a simple way and there is no need to have any previous knowledge about the electromyogram. Moreover, no additional visual information that may distract the user is provided apart from the target hand gesture. There are two bars (one for opening and one for hand closing) of variable length (proportional to the muscle activation) and two colors (exceed or not the predefined threshold). Thus, evidence has been found of the effectiveness on the implemented EMG-based visual feedback but cannot be applied to other types of feedback.

## 5. Conclusions

The incorporation of a real-time easily understandable EMG-based visual feedback enhances the performance of the subjects. This feedback allows the subjects to monitor their muscle activation in real time and, thus, modulate the exerted force. In contrast, kinesthetic feedback does not improve performance due to lag times and eliminates the positive influence of the EMG-based visual feedback if both are present. EMG-based visual feedback can be very useful in the learning stage so that the user can learn more quickly how to modulate their muscle activation so that the rehabilitation robot moves according to their intention. This may result in an improvement of the motivation of the patient for the rehabilitation process when using assistive robotic platforms.

Future work should be focused on people with neurological impairments to verify the results presented in this manuscript from a healthy population sample. On the other hand, the EMG is visually fed back in a simple manner, only using two variable length bars. Hence, further studies should be undertaken to confirm whether different kinds of EMG feedback have the same positive impact on the users’ performance and to determine its influence when used in combination with other virtual objects (e.g., in exergames based on virtual reality).

## Figures and Tables

**Table 1:** Test configuration.

	Kinesthetic Feedback	EMG-Based Visual Feedback
Test A	(✓)	(✓)
Test B	(✓)	(x)
Test C	(x)	(✓)
Test D	(x)	(x)

**Table 2:** Multifactorial additive ANOVA results of the L2 distances.

		Df	Sum Sq.	Mean Sq.	F Value	Pr (>F)
Test	*	3	3.366	1.1221	4.028	0.0124
Order		3	1.037	0.3456	1.241	0.3054
Individual	***	17	20.933	1.2313	4.420	2.43 × 10^−5^
Residuals		48	13.373	0.2786		

*** Denotes significance at the (<0.001) level and * at the (<0.5) level.

**Table 3:** Results of Duncan’s multiple range test.

	Test A	Test B	Test C
Test B	0.8775	-	-
Test C	0.0497 *	0.0412 *	-
Test D	0.3557	0.3121	0.2451

* Denotes significance at the (<0.5) level.

## Data Availability

The data analyzed in this study along with the code for statistical analysis are openly available in https://github.com/itap-robotica-medica/influence_of_visual_feedback (access on 31 January 2023).
